# Measuring oral health during pregnancy: sensitivity and specificity of a maternal oral screening (MOS) tool

**DOI:** 10.1186/s12884-016-1140-4

**Published:** 2016-11-09

**Authors:** Ajesh George, Hannah G. Dahlen, Anthony Blinkhorn, Shilpi Ajwani, Sameer Bhole, Sharon Ellis, Anthony Yeo, Emma Elcombe, Ayesha Sadozai, Maree Johnson

**Affiliations:** 1 Collaboration for Oral Health Outcomes, Research Translation and Evaluation (COHORTE) Research Group, Western Sydney University, South Western Sydney Local Health District, Ingham Institute Applied Medical Research, University of Sydney, Liverpool BC, Locked Bag 7103, Liverpool, NSW 1871 Australia; 2 School of Nursing & Midwifery, Western Sydney University, Ingham Institute Applied Medical Research, Parramatta, 2150 Australia; 3 Faculty of Dentistry, University of Sydney, Sydney, 2006 Australia; 4 Sydney Local Health District Oral Health Services, Sydney Dental Hospital, University of Sydney, Sydney, 2010 Australia; 5 Antenatal Services, Camden and Campbelltown Hospitals, South Western Sydney Local Health District, Campbelltown, 2560 Australia; 6 School of Nursing & Midwifery, Western Sydney University, Parramatta, 2150 Australia; 7 Western Sydney University, University of New South Wales, Ingham Institute Applied Medical Research, Liverpool, 2170 Australia; 8 Centre for Applied Nursing Research, Western Sydney University, South Western Sydney Local Health District, Ingham Institute Applied Medical Research, Liverpool, 1871 Australia; 9 Faculty of Health Sciences, Australian Catholic University, Ingham Institute Applied Medical Research, Sydney, 2060 Australia

**Keywords:** Oral health, Pregnancy, Midwives, Antenatal care, Prenatal care, Validation

## Abstract

**Background:**

Midwives can play a key role in promoting the oral health of pregnant women and assessing their oral health status. A maternal oral assessment tool (MOS) was developed and pilot tested by the study investigators to assist midwives in this role and the results were promising. The aim of this study was to undertake further sensitivity and specificity assessment of the MOS tool using two-comparison approaches- the longer oral health screening tool known as the Oral Health Impact Profile (OHIP-14) and an oral assessment by trained study dentists.

**Methods:**

Pregnant women were recruited for this study as part of a larger randomised controlled trial of a Midwifery Initiated Oral Health (MIOH) program. Pregnant women completed the MOS and OHIP-14 as part of their initial assessment undertaken by 38 trained and accredited midwives. A dental assessment was conducted for all women in the intervention group using three trained study dentists with high inter rater reliability.

**Results:**

Two hundred and eleven pregnant women participated in the validation of the MOS tool. Results from both approaches found the MOS tool to have high sensitivity, correctly identifying 88–94 % of women at risk of poor dental health, and low specificity (14–21 %).

**Conclusions:**

This study has shown that the MOS tool can be successfully implemented by midwives during a woman’s first antenatal visit and can identify up to 94 % of women at risk of poor oral health and needing a dental referral. The tool has the potential to be transferable to other antenatal care providers and could be incorporated into hospital obstetric database systems.

**Trial registration number:**

ACTRN12612001271897, 6^th^ Dec 2012, retrospectively registered.

**Electronic supplementary material:**

The online version of this article (doi:10.1186/s12884-016-1140-4) contains supplementary material, which is available to authorized users.

## Background

Population oral health experts within Australian and internationally have developed guidelines [1–4] that emphasise the importance of oral health screening for women during pregnancy. Pregnancy increases the risk of oral health problems (periodontal disease and tooth decay) due to physiological changes associated with pregnancy [5, 6]. In addition, early screening of women for oral health problems, provides an opportunity for women to learn about how to prevent decay for themselves and also for their future or existing children [1, 7]. The initial comprehensive review of women’s health is conducted at the antenatal clinic by midwives for most Australian women [8]. This provides a unique opportunity to assess and intervene to improve the oral health of women and their children. The potential for midwives and other antenatal care providers to also undertake an assessment of women’s health at that initial visit (primary care settings) has been supported in other studies [3, 4, 9]. The availability of a valid, simple to administer, oral health assessment tool with demonstrated sensitivity and specificity, would facilitate the identification of women with oral health problems and potentiate early dental interventions through referral during the optimal treatment time of the first trimester [10, 11].

Concerns raised about oral health during pregnancy go beyond dental caries. Systematic reviews have shown a positive association between adverse pregnancy outcomes such as preterm delivery and periodontal disease, although a cause and effect relationship has not been established (11, 12). Several trials have been conducted within Australia to intervene during pregnancy in an attempt to reduce the incidence of adverse birth outcomes such as the SMILE study [12] and the Midwifery Initiated Oral Health (MIOH) trial [13]. As part of the MIOH Trial we have included several instruments, and undertaken oral health assessments by dentists to determine the sensitivity and specificity of the Maternal Oral Screening (MOS) Tool.

The MOS tool was developed following a comprehensive literature review of screening items for oral health during pregnancy [10]. The result was a preferred two-item tool: item 1 referring to commonly reported dental problem during pregnancy and those caused by oral diseases [10, 14]; item 2 referring to how often the woman has seen a dentist in the last 12 months [10, 14]. A total score of ≥1 (having a dental problem or not seen a dentist in the previous 12 months) indicated that women were at risk of poor oral health and required a referral to a dentist. A visual inspection was optional for confirming the presence of dental problems and was only undertaken if a problem was identified by the pregnant women (See Table 1 below). An important part of the development process was the measurement of the tools sensitivity and specificity which indicates whether a patient will be screened in for an intervention (in this case dental treatment) or screened out (in this case having good oral health). A tool that allows for both situations to be considered with appropriate proportions is always an aspiration, however, in some clinical situations this may not always be possible [15]. Therefore the emphasis is often placed on whether the tool captures populations at risk of the condition, in this case women with poor oral health. Clinical evaluation of sensitivity and specificity is frequently used as the gold standard in clinical conditions where clinical assessment is a key diagnostic step [16]. We had previously pilot tested the MOS tool and found that it was promising in a sample of 56 women [10]. The pilot results suggested that the tool was acceptable to pregnant women and midwives and was sensitive (98 %) to identifying dental problems and facilitating dental referrals. However, further evaluation of the tool in a larger sample was warranted.

**Table 1:** Maternal Oral Screening tool for antenatal clinics

The maternal oral screening tool
Item 1. Do you have bleeding gums, swelling, sensitive teeth, loose teeth, holes in your teeth, broken teeth, toothache or any other problems in your mouth? Yes □ (1) No □ (0) If yes, visual inspection of oral cavity (optional to confirm Item 1) Item 2. Have you seen a dentist in the last 12 months? Yes □ (0) No □ (1)
Items 1 and 2 are scored either 0 or 1. Participants with a total score ≥ 1 are referred for a dental check-up.

### Aim

This study sought to undertake sensitivity and specificity assessment of the maternal oral health screening tool using two comparison approaches- the 14 item oral health screening tool known as the Oral Health Impact Profile (OHIP-14) and a clinical oral assessment by trained study dentists.

## Methods

This study was undertaken as part of an existing multicenter randomized controlled trial that is evaluating the effectiveness of a midwifery initiated oral health program in improving the uptake of dental services, oral health knowledge, quality of life and oral health status of pregnant women (Trial ID ACTRN12612001271897). At the initial assessment, pregnant women, undergo screening and referral (where appropriate) by appropriately accredited midwives [17].

### Sample and setting

The pregnant women were recruited from three large antenatal clinics in Sydney. The eligibility criteria for the MIOH trial have been reported elsewhere [13] and included low risk women with a single pregnancy (more than 12 and less than 20 weeks gestation). These women were screened at their initial antenatal appointment and provided dental referrals by accredited midwives who had completed the MIOH education program [17, 18]. One group of women in the trial was referred to study dentists for a clinical examination regardless of whether they were screened to be at risk of poor oral health. These women formed the study sample for the testing of the MOS tool.

### Characteristics of women attending the three antenatal clinics

Two hundred and eleven participants were recruited for this study. The majority of participants were Australian born (*n* = 128, 61 %) with a mean age of 29 years (SD = 5.7, range = 18–43 years). Most women were multiparous (65 %), partnered (81 %) and in their second trimester (*n* = 387, 92 %). The highest level of education recorded was university (18 %) followed by trade school (35 %). Most participants were not working (56 %) or working part time (22 %). More than 40 % of participants (*n* = 83) had a combined annual household income of less than $60,000 and a high proportion (50 %) lived in areas of low socio-economic advantage (lowest Socio-Economic Index for Area quintile as measured through the Australian Bureau of Statistics).

### Characteristics of midwives undertaking the screening and referral of women

Thirty-eight midwives successfully completed the MIOH education program and undertook the screening and referral of the pregnant women. The midwives had a mean age of 43.4 years (SD 11.5) and an average of 15.8 years of clinical practice experience (SD 11.7). Nearly half the midwives (47.4 %) had postgraduate qualifications.

### OHIP-14

The OHIP-14 is a subjective measure of oral health that has been found to be a precise, valid and reliable instrument (α = 0.88) [19]. It has been used previously as a ‘gold standard’ measure for validating other oral assessment tools [20]. OHIP-14 contains 14 questions assessed on a 5-point Likert scale and the total scores range from 0 to 56 with higher scores indicating poorer oral health. This tool was developed based on a conceptual model defined by Locker [21] focusing on seven domains- functional limitation, physical pain, psychological discomfort, physical disability, psychological disability, social disability and handicap (see Additional file 1).

### Clinical examination by study dentists

Three experienced dentists were recruited to the study from health services where the study was conducted. Several reliable measures were used to assess the extent of dental problems in pregnant women. These included the decay, missing and filled teeth (DMFT) index [22] and the periodontal screening and reporting (PSR) index (24). The DMFT index identified whether decay was present while the PSR index (scoring 0–4) assessed the health of the gums with a score of two and above indicating gum disease requiring treatment [23]. Pregnant women were determined to have poor oral health if they had any dental decay and a PSR score of greater than two. One of the investigators (SA) undertook to train all dentists on a standard assessment and intervention protocol. The dentists were assessed on five patients for various measures with >80 % inter-rater reliability in the key study measures (DMFT and PSR).

### Procedure

The items for the MOS and OHIP-14 were included in the initial assessment undertaken by the midwife with the consenting woman presenting to the antenatal clinic. The data collection procedures for the MIOH Trial are more fully described within the protocol [13]. The dental assessment was conducted for all women in the intervention group that had access to the study dentists [13]. Ethical approval was obtained from the Human Research and Ethics Committees of Sydney Local Health District (reference no HREC/11/CRGH/28) and Western Sydney University (reference no H9709). Written consent to participate in the trial was obtained from women attending the antenatal clinic at the initial presenting visit.

### Data management and analysis

Statistical analysis was completed using SPSS v22. Descriptive statistics were used to describe the demographic information of the sample population. The relationships between the screening tools were assessed using chi-squared analyses after variable dichotomisation. The level of significance (α) was set at 0.05. Sensitivity, specificity, positive predictive value (PPV) and negative predictive value (NPV) were computed using conditional probabilities based on a two-way table. Confidence intervals were determined using the central limit theorem formulation.

## Results

### MOS tool

In total, an average of 67 % (*n* = 141) of women reported having a current problem or concern with their teeth, gums or mouth, and 39 % reported seeing a dentist in the previous 12 months. Using the screening criteria (Table 1), 86 % of women were deemed at risk of poor oral health (Table 2).

**Table 2:** Women’s responses to the MOS tool, OHIP-14 and Dental Assessment

	No. (%)
Maternal Oral Screening Tool (*n* = 207)	
Item 1: Do you have problems in your mouth? [Yes]	141 (66.8 %)
Item 2: Have you seen a dentist in the last 12 months? [No]	128 (60.7 %)
‘At risk’ of poor oral health^1^	181 (85.8 %)
OHIP-14 (Self-report) (*n* = 207)	
Score > 4	90 (43.5 %)
Dental Assessment (*n* = 131)	
Any tooth decay detected? [Yes]	81 (62 %)
PSR rating ≥ 2 [Yes]	118 (90 %)
Women with poor oral health^2^	74 (56 %)

*PSR* Periodontal screening and recording index

^1^Participants considered ‘At risk’ of poor oral health if they reported a problem with their teeth, gums or mouth OR if they had not seen a dentist in the previous 12 months, *n* = 207

^2^Participants considered to have poor oral health if they had dental decay detected and had a PSR score of ≥ 2, *n* = 131

### Approach 1 –OHIP- 14

A total of 207 (98 %) participants satisfactorily completed the OHIP-14. The mean OHIP-14 score was 6.8 (SD = 8.6, median = 4.0, range 0–38). As cut-off scores for identifying patients at risk of poor oral health has not been established with the OHIP-14, the total score for each participant was dichotomised using the median split as described by Locker et al. [24] categorised as either ‘At risk’ (score greater than 4) or ‘Not at risk’ (score 0 to 4) (Table 2).

### Approach 2 - dental assessment

Of the 207 participants referred, 131 women completed the dental assessments. Analysis of these data found 90 % (*n* = 118) of women had a PSR rating of two or more, 62 % (*n* = 81) had dental decay, and 56 % (*n* = 74) of women had both of these and were considered to have poor oral health.

### Sensitivity and specificity

The MOS data was assessed against two gold standard measures of oral health; the 14 item OHIP questionnaire and a clinical oral health check. Both analyses found the MOS tool to have high sensitivity (88–93 %) for detecting women at risk of poor oral health. More specifically, analysis of the MOS tool against the OHIP-14 found that Item 1 of the MOS gave the highest sensitivity (82.2 %) while Item 2 gave the highest specificity (56.4 %). When items 1 and 2 were combined the sensitivity increased to 93.3 % while the specificity decreased (Table 3). A similar pattern of results was seen when the MOS tool was compared to the Clinical oral assessment with the highest sensitivity (87.8 %) being achieved by combining items 1 and 2. Overall, combining items 1 and 2 of the MOS tool resulted in relatively low specificities (Dental assessment = 14.0 % and OHIP-14 = 20.5 %).

**Table 3:** Sensitivity and specificity of Maternal Oral Screening Tool compared with OHIP-14 and Dental Assessment

	MOS - Item 1	MOS - Item 2	Combined
	% (95 % CI)	% (95 % CI)	% (95 % CI)
OHIP-14 (Self-report)^1^ (*n* = 207)			
Sensitivity	82.2 (74.3–89.1)	32.2 (22.6–40.7)	93.3 (88.2–97.9)
Specificity	46.2 (37.1–55.2)	56.4 (47.4–65.4)	20.5 (13.2–27.8)
PPV	54.0 (45.7–62.4)	36.3 (25.7–46.8)	47.5 (40.1–54.8)
NPV	77.1 (67.3–87.0)	52.0 (43.3–60.7)	80.0 (65.7–94.3)
Dental assessment (PSR > 2 and Decay present) (*n* = 131)			
Sensitivity	70.3 (59.9–82.1)	41.9 (30.7–54.7)	87.8 (80.4–96.3)
Specificity	29.8 (17.9–41.7)	68.4 (56.4–80.5)	14.0 (5.0–23.1)
PPV	56.5 (46.4–66.7)	63.3 (49.8–76.8)	57.0 (47.9–66.1)
NPV	43.6 (28.0–59.2)	47.6 (36.8–58.4)	47.1 (23.3–70.8)

^1^Item 1 = Do you currently have any problems with your teeth Gums or mouth?, Item 2 = Have you had a dental check up in the last 12 months?

*CI* Confidence interval, *PPN* Positive predictive value, *NPV* Negative predictive value

## Discussion

The focus of this study was to further validate a maternal oral health screening tool for pregnant women. Developing such a tool is important because of the high prevalence of poor oral health among pregnant women and its impact on maternal and infant outcomes (5). Further, current guidelines advocate the need for all antenatal care providers to undertake oral health education, assessment and referral during early pregnancy (1–3). Initial piloting of the MOS tool suggested that it was easy to administer by midwives and had good sensitivity in identifying dental problems in pregnant women and facilitating referrals (15). However, the pilot study had limited sample size and further testing of the sensitivity and specificity was warranted.

This study used a two staged approach using a larger sample size to validate the MOS tool. Current practice during the first antenatal visit involves midwives assessing multiple health areas at one time, thus consideration of time constraints was an important factor in assessing oral health during this visit (25). This was one of the main reasons why only three items were considered during the development phase of the tool (15), only two of which were used in this study. The study results reiterate earlier suggestions that the 2-item tool (which is part of the MIOH education program) was easy to administer by midwives and comprehend by pregnant women (16, 18, 19).

Validation of the MOS tool (2-item) showed high sensitivity across both gold standards (88–97 %) which is similar to results of the pilot study (75–98 %). This combined with moderated NPV’s of 47–80 % confirm that the MOS tool is reliable and able to correctly identify the majority of women in need of a dental referral. The specificity of the tool remained low even with increased sample size (14–19 %), however when interpreting this value consideration must be given to the purpose to the tool. In this situation correct identification of women at risk of poor oral health, compared to identifying those with good oral health, is of paramount importance especially considering that it is recommended that all women should see a dentist early in their pregnancy even if they are unaware of any dental problems (1, 12). The positive predictive value of the MOS tool indicates that around 50 % of the women referred for a dental assessment, will in fact be in need of dental care. In addition it should be noted that low specificity values are not uncommon in oral health screening tools with values ranging from 2.9 to 37.5 % observed in studies addressing oral health in early childhood (27) and HIV patients (26).

The study reinforces earlier suggestions (15) that the MOS tool can be easily administered by midwives. The fact that the education that accompanies the assessment tool has been successfully implemented in other states in Australia (19) indicates a level of tool transference. In the future this tool could be used by other antenatal care providers as an assessment tool. In addition, there is no restriction on this tool being used solely by midwives, therefore other health care professionals who have contact with mothers early in the antenatal period may also be able to successfully undergo the training and administered the MOS tool. Further the tool could also be incorporated into existing obstetric systems to increase the number women captured/screened across the country.

Although the tool has low specificity, in the current Australian system, where we have strict eligibility criteria’s for public dental services (28) as opposed to a free universal dental scheme for pregnant women, the impact of over referral will be mostly borne by the individual rather than the government. The potential cost benefits however is not to be disregarded; the encouragement of women to access dental services earlier during pregnancy is likely to have flow on health benefits, improving maternal health and quality of life (29), child oral health (30) and potentially birth outcomes (31) which ultimately could reduce the overall cost burden to society.

Lastly, it should be noted that although the OHIP- 14 is frequently used as a gold standard for measures in a variety of populations such as older people [19, 25] and HIV patients [20] it may not be as appropriate for this population. Only 6 of the 14 OHIP items were scored frequently >0 in the study population. These items were related to the physical pain, psychological discomfort and psychological disability domains of the OHIP-14. As the OHIP tool is derived from the conceptual understanding of Locker et al. [21] focusing on disability, the remaining domains and related items may not be as suitable for this sample of healthy women. Further studies should be conducted to develop or modify the OHIP-14 for this population of predominantly young healthy women. Similarly as suggested by Locker et al. [24] longitudinal studies should be undertaken to identify the ability of the OHIP tool to measure changes over time.

### Limitations

This study was undertaken in South West Sydney and the findings may deliver differing results in other populations of pregnant women from higher socio-economic backgrounds, or indeed from other countries. Similarly this study has focused on only one aspect of psychometric assessment of a tool and other aspects such as construct validity was not undertaken within this sample. We also note that although the OHIP-14 was used as a gold standard, similar results in terms of sensitivity and specificity were also demonstrated with clinical examination by dentists.

## Conclusion

The MOS (2-item) provides a sensitive screening tool that is easy to use by midwives and potentially other antenatal care providers including general practitioners. The items are easily administered and provide an opportunity to raise awareness with women about the importance of oral health to their overall health as well as the health of the unborn fetus. This simple initial screening approach conducted by a non-oral health professional, with specialized training, complements the service provided by dentists where more thorough examination of oral health risk can be undertaken. Further studies should examine the potential for the OHIP-14 or other modifications of the OHIP to more appropriately target items of relevance for young healthy pregnant women.

## Additional file

Additional file 1: Oral Health Impact Profile (OHIP 14) Questionnaire. (DOCX 15 kb)

## Abbreviations

DMFT: Decay, missing and filled teeth
MIOH: Midwifery Initiated Oral Health program
MOS: Maternal oral assessment tool
NPV: Negative predictive value
OHIP-14: Oral Health Impact Profile
PPV: Positive predictive value
PSR: Periodontal screening and reporting
