# Supplementary material for: Measuring oral health during pregnancy: sensitivity and specificity of a maternal oral screening (MOS) tool
Source: BMC Pregnancy Childbirth. 2016 Nov 9;16:347. doi: 10.1186/s12884-016-1140-4 (PMC5103484; doi:10.1186/s12884-016-1140-4)
Supplement: Additional file 1: — Oral Health Impact Profile (OHIP 14) Questionnaire. (DOCX 15 kb) [file 12884_2016_1140_MOESM1_ESM.docx]

**Oral Health Impact Profile (OHIP 14) Questionnaire**

| Question | Never | Hardly ever | Occasi  onally | Fairly often | Very often |
| --- | --- | --- | --- | --- | --- |
| 1. Have you had trouble pronouncing any words because of problems with your teeth, mouth or dentures? |  |  |  |  |  |
| 1. Have you felt that your sense of taste has worsened because of problems with your teeth, mouth or dentures? |  |  |  |  |  |
| 1. Have you had painful aching in your mouth? |  |  |  |  |  |
| 1. Have you found it uncomfortable to eat any foods because of problems with your teeth, mouth or dentures? |  |  |  |  |  |
| 1. Have you been self-conscious because of your teeth, mouth or dentures? |  |  |  |  |  |
| 1. Have you felt tense because of problems with your teeth, mouth or dentures? |  |  |  |  |  |
| 1. Has your diet been unsatisfactory because of problems with your teeth, mouth or dentures? |  |  |  |  |  |
| 1. Have you had to interrupt meals because of problems with your teeth, mouth or dentures? |  |  |  |  |  |
| 1. Have you found it difficult to relax because of problems with your teeth, mouth or dentures? |  |  |  |  |  |
| 1. Have you been a bit embarrassed because of problems with your teeth, mouth or dentures? |  |  |  |  |  |
| 1. Have you been a bit irritable with other people because of problems with your teeth, mouth or dentures? |  |  |  |  |  |
| 1. Have you had difficulty doing your usual jobs because of problems with your teeth, mouth or dentures? |  |  |  |  |  |
| 1. Have you felt that life in general was less satisfying because of problems with your teeth, mouth or dentures? |  |  |  |  |  |
| 1. Have you been totally unable to function because of problems with your teeth, mouth or dentures? |  |  |  |  |  |
